# Antihyperlipidemic Effect of a Polyherbal Mixture in Streptozotocin-Induced Diabetic Rats

**DOI:** 10.1155/2013/675759

**Published:** 2013-12-05

**Authors:** Ahmad Ghorbani, Reza Shafiee-Nick, Hassan Rakhshandeh, Abasalt Borji

**Affiliations:** ^1^Pharmacological Research Center of Medicinal Plants, School of Medicine, Mashhad University of Medical Sciences, Mashhad 9177948564, Iran; ^2^Department of Pharmacology, School of Medicine, Mashhad University of Medical Sciences, Mashhad 9177948564, Iran; ^3^Neyshabur University of Medical Sciences, Neyshabur 93186-14139, Iran

## Abstract

The effects of a polyherbal mixture containing *Allium sativum*, *Cinnamomum zeylanicum*, *Citrullus colocynthis*, *Juglans regia*, *Nigella sativa*, *Olea europaea*, *Punica granatum*, *Salvia officinalis*, *Teucrium polium*, *Trigonella foenum*, *Urtica dioica*, and *Vaccinium arctostaphylos* were tested on biochemical parameters in diabetic rats. The animals were randomized into three groups: (1) normal control, (2) diabetic control, and (3) diabetic rats which received diet containing 15% (w/w) of this mixture for 4 weeks. Diabetes was induced by intraperitoneal injection of streptozotocin (55 mg/kg). At the end of experiment, the mixture had no significant effect on serum hepatic enzymes, aspartate aminotransferase, and alanine aminotransferase activities. However, the level of fasting blood glucose, water intake, and urine output in treated group was lower than that in diabetic control rats (*P* < 0.01). Also, the levels of triglyceride and total cholesterol in polyherbal mixture treated rats were significantly lower than those in diabetic control group (*P* < 0.05). Our results demonstrated that this polyherbal mixture has beneficial effects on blood glucose and lipid profile and it has the potential to be used as a dietary supplement for the management of diabetes.

## 1. Introduction

Diabetes mellitus, a metabolic disease with manifestation of hyperglycemia and dyslipidemia, is still one of the most leading causes of death and disability. Over time, diabetes leads to serious complications such as nephropathy, retinopathy, neuropathy, stroke, and peripheral vascular diseases [1]. Currently, beside insulin, the most widely used medications for diabetes are insulin and the oral hypoglycemic drugs [2]. Although early onset manifestations of diabetes can be controlled by current antidiabetic drugs, late onset complications appear in many patients [3]. In addition, the clinical uses of the current drugs are usually accompanied with some adverse effects including abdominal discomfort, severe hypoglycemia, lactic acidosis, and peripheral edema [2]. Therefore, the search for new antidiabetic agents with more effectiveness and lesser side effects has continued.

Today, antidiabetic effects of several plants have been supported by results from animal studies or clinical trials. Among them, *Allium sativum*, *Cinnamomum zeylanicum*, *Citrullus colocynthis*, *Juglans regia*, *Nigella sativa*, *Olea europaea*, *Punica granatum*, *Salvia officinalis*, *Teucrium polium*, *Trigonella foenum*, *Urtica dioica*, and *Vaccinium arctostaphylos* are widely used as medicinal plants for management of diabetes [4, 5]. Several studies have shown that each one of these plants is effective in the decrease of plasma glucose and serum lipids in diabetes [6–18]. We hypothesized that a combination of them may have more beneficial effect on improving metabolic indexes. The present study is a part of a research effort to develop the best polyherbal mixture from these antidiabetic plants for management of glucose and lipid in diabetes. In our previous work, we observed that administration of a combination of hydroalcoholic extracts of six of them inhibited the progression of hyperglycemia and decreased serum lipids and hepatic enzyme activity in diabetic rats [19]. Since nonprocessed herbs are usually more tolerated than extracted or formulated products and patients prefer to consume plants as salad, spice, and so forth, in this work, we aimed to investigate the effect of a mixture of powders of the above mentioned twelve plants on glycemic and lipidemic status of diabetic rats.

## 2. Materials and Methods 

### 2.1. Preparation of Polyherbal Mixture

The air-dried *A. sativum* (cloves), *C. zeylanicum* (bark), *C. colocynthis* (fruit), *J. regia* (leaf), *N. sativa* (seeds), *O. europaea* (leaf),* P. granatum* (fruit),* S. officinalis* (areal parts), *T. polium* (areal parts), *T. foenum* (seeds), *U. dioica* (areal parts), and* V. arctostaphylos* (fruit) were powdered and mixed with ratio of 5%, 5%, 5%, 10%, 5%, 5%, 5%, 5%, 5%, 20%, 15%, and 15%, respectively. This polyherbal mixture (PHM) was added (15% w/w) to the standard pellets diet of animals in treatment group.

### 2.2. Animals

Male albino Wistar rats (280–330 g) were obtained from Laboratory Animals House, Mashhad Medical University (Iran) and housed in a room with controlled lighting (12 h light/12 h darkness) and temperature (22 ± 2°C). The animals were given standard pellets diet and water *ad libitum*. All animal procedures were done according to the ethical guidelines of the animal care of Shiraz University of Medical Sciences (Iran). To generate diabetes, some rats received a single dose (55 mg/kg, ip) of streptozotocin (STZ) (Enzo Life, USA). Induction of diabetes was confirmed by measuring fasting blood glucose (FBG) two days after STZ injection. Rats with FBG level of 250 mg/kg or higher were considered to be diabetic [20, 21].

### 2.3. Groups of Study

The animals were randomized into three groups: (1) normal control rats which were fed standard diet (*n* = 6), (2) diabetic control animals which were fed standard diet (*n* = 6), and (3) diabetic rats which received diet containing 15% (w/w) of PHM (*n* = 8). The treatment was initiated two days after STZ injection and continued for 4 weeks. At the end of the 30th day, the rats fasted 16 h and blood samples were collected from retroorbital sinus for biochemical measurements. At the end of the experiment, animals were placed in individual metabolic cages for urinary collection. After acclimatization (1 day), the 24 h urinary samples were collected from diabetic animals.

### 2.4. Biochemical Assays

Serum triglyceride and total cholesterol were evaluated with standard enzymatic colorimetric kits from Pars Azmun (Iran). Blood glucose was measured using glucose oxidase reagent (Ziest Chem Diagnostics, Iran). Serum aspartate aminotransferase (AST) and alanine aminotransferase (ALT) activities were evaluated with colorimetric methods by commercially available kits (Pars Azmun, Iran).

### 2.5. Statistical Analysis

Analysis of changes from baseline was performed by paired *t*-test within groups. Intergroup comparison was done by one-way ANOVA with Tukey's posthoc test. Results showing *P* values less than 0.05 were considered significant.

## 3. Results

### 3.1. Effect of PHM on Blood Glucose

**Figure 1:**
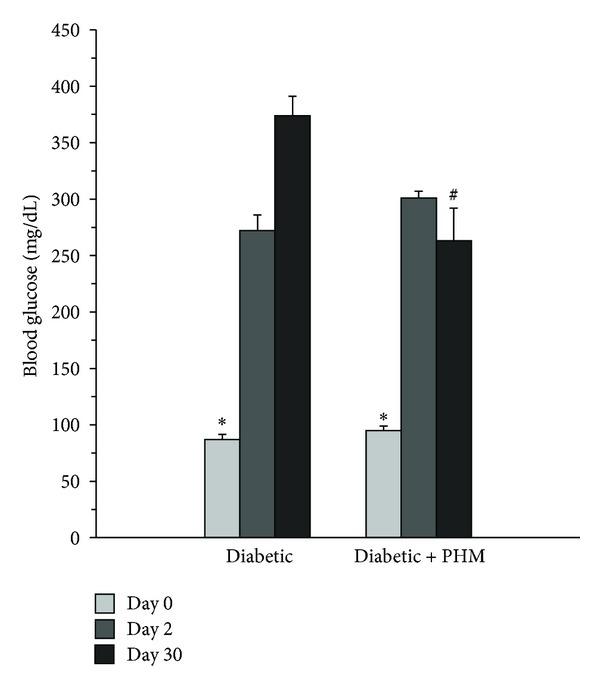
Effects of the polyherbal mixture (PHM) on blood glucose. The animals in group of diabetic + PHM received PHM for 4 weeks. *P < 0.01 compared with day 2 and day 30 at the corresponding group. ^#^ *P* < 0.01 compared with the corresponding value in diabetic control group. The data are expressed as mean ± SEM for eight (diabetic + PHM) or six (normal and diabetic control) rats.

As shown in Figure 1, before injection of STZ, FBG levels of both groups were statistically not different from each other. At day 2, administration of STZ led to an approximately 3-fold increase in FBG level. The diabetic rats in control group showed further increase of FBG at the end of experiment (374 ± 17 mg/dL in day 30 versus 272 ± 14 mg/dL in day 2, *P* < 0.01). However, administration of PHM to diabetic rats blocked the increase of blood glucose. At day 30, the level of FBG in this group was 263 ± 29 mg/dL which was significantly lower than that in diabetic control rats (*P* < 0.01).

### 3.2. Effect of PHM on Body Weight, Water Intake, and Urine Output

During experiment, both diabetic control and diabetic PHM treated groups exhibited a significant reduction in their body weight. At day 30, the weight reduction reached to 27% (*P* < 0.001 versus day 0) and 25% (*P* < 0.001 versus day 0) for control and PHM treated animals, respectively (Table 1). Prior to diabetes induction, the level of water intake was not significantly different between three groups. However, after STZ administration, there was a significant increase in the levels of water intake in both groups of diabetic rats. Although the polydipsia condition was evident during the treatment period, the level of water intake in PHM group was significantly (*P* < 0.001) lower than that in diabetic control group. Also, at the end of the experiment, the PHM-fed diabetic rats showed an improvement in polyurea state and the excretion of urine was significantly lower than that of diabetic control group (30 ± 3 mL/24 h versus 72 ± 9 mL/24 h, *P* < 0.01).

### 3.3. Effect of PHM on Serum Lipids

**Figure 2:**
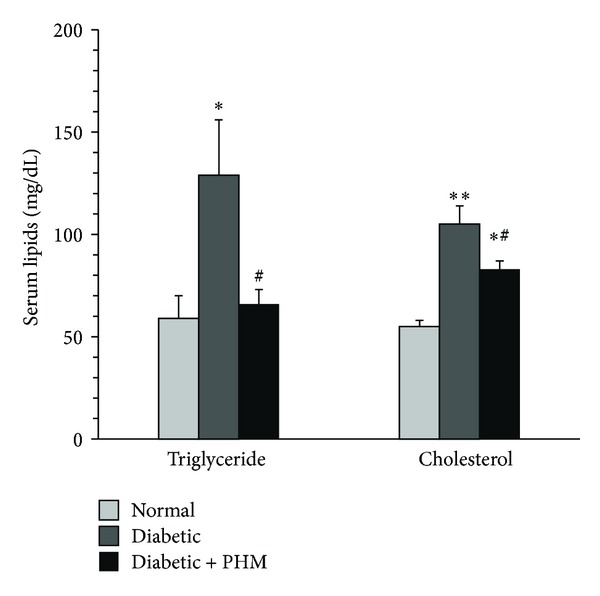
Effects of the polyherbal mixture (PHM) on the levels of plasma lipids. The animals in group of diabetic + PHM received PHM for 4 weeks. *P < 0.05 versus normal rats. **P < 0.01 versus normal rats. ^#^ *P* < 0.05 versus diabetic control. The data are expressed as mean ± SEM for eight (diabetic + PHM) or six (normal and diabetic control) rats.

Figure 2 shows the level of serum lipids in study groups. There was a significant elevation in the level of triglyceride (129 ± 27 mg/dL versus 59 ± 11 mg/dL, *P* < 0.05) and total cholesterol (105 ± 9 mg/dL versus 55 ± 3 mg/dL, *P* < 0.01) in diabetic control rats as compared with normal group. The PHM was found to be effective in decreasing serum lipids. The levels of triglyceride and total cholesterol in PHM treated group were 66 ± 27 mg/dL (*P* < 0.05 versus diabetic control group) and 83 ± 4 mg/dL (*P* < 0.05 versus diabetic control group), respectively.

### 3.4. Effect of PHM on Hepatic Enzymes

**Figure 3:**
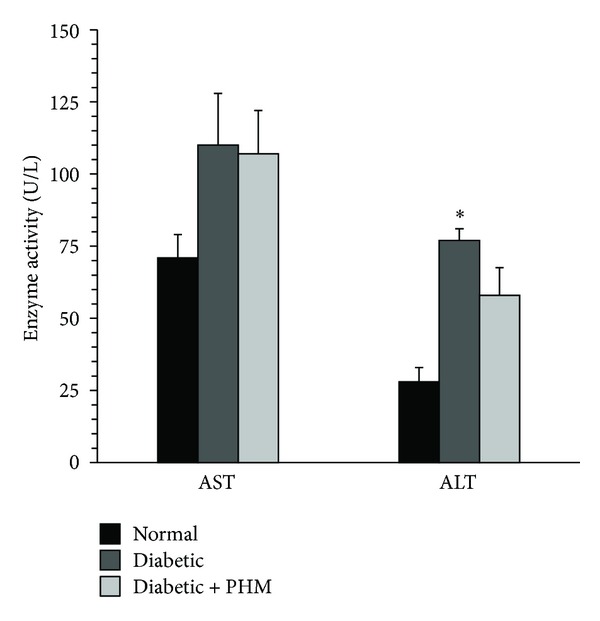
Effects of the polyherbal mixture (PHM) on serum aspartate aminotransferase (AST) and alanine aminotransferase (ALT) activities. The animals in group of diabetic + PHM received PHM for 4 weeks. *P < 0.05 versus normal control rats. The data are expressed as mean ± SEM for eight (diabetic + PHM) or six (normal and diabetic control) rats.

At day 30, diabetic control rats showed higher AST (110 ± 18 versus 71 ± 8 U/L) and ALT (77 ± 4 versus 28 ± 5 U/L, *P* < 0.05) activity than that of normal group, suggesting hepatic dysfunction in these animals. The level of AST and ALT activity in PHM treated groups was 107 ± 15 U/L and 58 ± 10 U/L, respectively, which was not significantly different from that of diabetic control rats (Figure 3).

## 4. Discussion

Although numerous herbs have been suggested for the treatment of diabetes, but at present no one could completely treat diabetic patients. One approach for the development of an effective phytochemical compound can be mixing a number of hypoglycemic and hypolipidemic herbs to produce more potent antidiabetic agent. The present work was a part of a research effort to find the best mixture from some medicinal plants which their hypoglycemic and hypolipidemic effects were supported by several studies. Here, we investigated the effect of a diet containing mixture of twelve medicinal plants on glycemic and lipidemic status of diabetic rats. Although this mixture failed to completely restore STZ-induced hyperglycemia and had no effect on weight reduction, it significantly prevented further elevation of blood sugar and improved the polydipsia and polyurea. This beneficial effect on glycemic status is expected to happen as antihyperglycemic effect of all twelve plants forming PHM has been confirmed with repeated studies [6–16, 22, 23]. Antihyperglycemic effect of medicinal plants is achieved by different mechanisms including decreasing glucose absorption from intestine, enhancing insulin secretion from beta cells, increasing glucose uptake by tissues, inhibiting glucose production in liver, and increasing pancreatic tissue regeneration and/or presence of insulin-like agents in plants [24–27]. Regarding PHM, inhibitory effect of *T. foenum* on intestinal glucose absorption and the beneficial effects of *J. regia* and *T. polium* on regeneration of pancreatic islets were reported [28–30]. Also, it has been demonstrated that *N. sativa* and *S. officinalis* decrease hepatic glucose production through inhibition of gluconeogenic enzymes [31, 32]. Furthermore, the insulin secretory effects of *C. colocynthis*, *O. europaea*, *T. foenum*, *V. arctostaphylos*, and *U. dioica* were shown *in vitro* in the isolated pancreas or islets [16, 17, 33–35].

Diabetes is often associated with dyslipidemia, a main risk factor of cardiovascular diseases. Therefore, the levels of serum triglyceride and cholesterol are usually elevated in diabetic patients [36]. In our study, PHM could decrease serum triglyceride level and attenuate hypercholesterolemia. The hypolipidemic action of PHM is in agreement with earlier studies that reported that *A. sativum*, *C. colocynthis*, *C. zeylanicum*, *J. regia*,* N. sativa*, *S. officinalis*, *P. granatum*, *T. foenum,* and *T. polium* decrease the levels of serum triglyceride and cholesterol in diabetic subjects [6, 7, 9, 12, 14, 37–39]. Also, in our previous work, we showed that *T. foenum* led to a significant reduction in lipid droplet accumulation in adipocytes [40].

Elevation of ALT and AST enzyme activities is considered as an evidence for hepatic damage. An increase of these enzyme activities is also associated with fatty liver disease and decreased hepatic insulin sensitivity in type 2 diabetes [41, 42]. According to the reports of previous studies, *A. sativum*, *J. regia*, *S. officinalis*, *O. europaea*, *T. foenum,* and *T. polium* could decrease the ALT and AST levels in diabetic rats [7, 8, 37, 43–45]. On the other hand, there are some reports that chronic administration of* T. polium* results in elevation in plasma levels of liver enzymes [15, 46]. In our study, the PHM has no significant effect on ALT and AST activity. Therefore, consumption of this polyherbal mixture accompanied with no hepatoprotective or hepatotoxic activity.

Taken together, our results demonstrated that PHM has beneficial effects on blood glucose and lipid profile of diabetic rats. In our previous work, we observed approximately the same level of antihyperglycemic and hypolipidemic effect with administration of a polyherbal mixture made from the combination of macerated and Soxhlet (hydroalcoholic) extracts of *A. sativum*, *C. zeylanicum*, *N. sativa*, *P. granatum*, *S. officinalis, *and *T. polium* [19]. Therefore, we did not find any favorable effects on diabetes when the powders of twelve plants were combined instead of giving a combination of hydroalcoholic extracts of six of these plants. Also, some studies reported more antihyperglycemic effect when the extracted or formulated products of herbs especially *T. foenum* were given individually [28]. However, it should be considered that when they are administrated individually, the antidiabetic effect was achieved usually with high doses of the plant extract which may be accompanied with unpleasant effects in the body [47]. Therefore, we still believed that the PHM, which was constructed simply from mixing plant powders without the need for extraction procedure, has the potential to be used as a dietary supplement for the management of diabetes, particularly diabetic dyslipidemia. Obviously, more study is needed to find a more potent polyherbal mixture from antidiabetic plants. Further, combination of active ingredients isolated from these plants may be a subject if interest in future perspective related to diabetes management.

## Figures and Tables

**Table 1:** Effects of the polyherbal mixture (PHM) on body weight and water intake. The animals in group of diabetic + PHM received PHM for 4 weeks.

Animal groups	Body weight (gram)		Water intake (mL/24 h)			Urine (mL/24 h)
	Day 0	Day 30	Day 0	Day 16	Day 30	Day 30
Normal control	311 ± 5	360 ± 7*	37 ± 3	50 ± 4^**∞**^	42 ± 2^**∞**^	—
Diabetic control	314 ± 6	230 ± 6^∗∗^	42 ± 2	137 ± 7^#^	143 ± 11^#^	72 ± 9
Diabetic + PHM	300 ± 6	223 ± 6**	40 ± 3	84 ± 2^#×^	100 ± 9^#×^	30 ± 3^×^

*P < 0.05 compared with day 0 in the corresponding group. **P < 0.001 compared with day 0 in the corresponding group. ^#^ *P* < 0.001 compared with the corresponding values at day 0. ^**∞**^ *P* < 0.001 compared with the corresponding values of diabetic and diabetic + PHM groups. ^×^ *P* < 0.01 compared with the corresponding values of diabetic group. The data are expressed as mean ± SEM for eight (diabetic + PHM) or six (normal and diabetic control) rats.
